# Supplementary material for: Diversity of Ladybird Beetles (Coleoptera: Coccinellidae) in Tenerife and La Gomera (Canary Islands): The Role of Size and Other Island Characteristics
Source: Insects. 2024 Aug 6;15(8):596. doi: 10.3390/insects15080596 (PMC11354425; doi:10.3390/insects15080596)
Supplement: Supplementary file 1 [file insects-15-00596-s001.zip › insects-3029040-supplementary.pdf]

**Supplement 1**. Collection sites of ladybird beetles on Tenerife and La Gomera.

| **Location** | **Coordinates** | **Altitude (m a.s.l.)** |
|---|---|---|
| **Tenerife** | | |
| 1 Adeje env. | 28°08'01"N 16°42'33"W | 535 |
| 2 Aeropuerto de Tenerife Sur | 28°02'59"N 16°34'40"W | 80 |
| 3 Aguamansa env. | 28°20'30"N 16°31'45"W | 1421 |
| 4 Afur env. | 28°33'11"N 16°14'56"W | 279 |
| 5 Area Recreativa Chio | 28°15'57"N 16°44'58"W | 1555 |
| 6 Area Recreativa Las Lajas | 28°11'22"N 16°39'58"W | 2093 |
| 7 Arico | 28°10'54"N 16°28'25"W | 350 |
| 8 Arona | 28°05'56"N 16°40'51"W | 624 |
| 9 Barranco de Acetenjo | 28°26'15"N 16°27'07"W | 591 |
| 10 Barranco de Ajeque | 28°21'50"N 16°52'56"W | 303 |
| 11 Barranco de la Torre | 28°21'23"N 16°52'27"W | 276 |
| 12 Buenavista del Norte | 28°21'39"N 16°52'11"W | 114 |
| 13 Chamorga | 28°34'36"N 16°09'11"W | 596 |
| 14 Chimiche | 28°07'53"N 16°31'55"W | 427 |
| 15 Chirche | 28°13'26"N 16°45'36"W | 976 |
| 16 Cruz del Carmen | 28°31'55"N 16°17'01"W | 972 |
| 17 Mirador de Cherfe | 28°17'58"N 16°49'26"W | 1060 |
| 18 El Bailadero | 28°33'01"N 16°12'11"W | 676 |
| 19 El Chorrillo | 28°24'48"N 16°19'54"W | 326 |
| 20 El Guincho | 28°01'35"N 16°36'47"W | 33 |
| 21 El Rosario | 28°24'56"N 16°19'20"W | 300 |
| 22 El Sauzal | 28°28'23"N 16°25'59"W | 441 |
| 23 Granadilla | 28°07'03"N 16°34'34"W | 637 |
| 24 Icor | 28°12'19"N 16°27'20"W | 330 |
| 25 La Caldera | 28°21'28"N 16°30'07"W | 1185 |
| 26 La Esperanza | 28°27'05"N 16°22'03"W | 904 |
| 27 La Orotava | 28°23'22"N 16°31'23"W | 374 |
| 28 La Sabinita | 28°10'50"N 16°29'46"W | 550 |
| 29 Las Eras | 28°12'05"N 16°25'51"W | 97 |
| 30 Las Galletas | 28°00'26"N 16°39'26"W | 3 |
| 31 Las Lagunetas | 28°25'07"N 16°24'37"W | 1407 |
| 32 Las Maretas | 28°05'50"N 16°28'54"W | 3 |
| 33 Las Portelas | 28°19'45"N 16°50'33"W | 726 |
| 34 Las Vegas | 28°08'49"N 16°32'49"W | 640 |
| 35 Los Batanes env. | 28°32'23"N 16°17'36"W | 580 |
| 36 Los Christianos | 28°02'58"N 16°42'48"W | 30 |
| 37 Los Pinos | 28°23'18"N 16°30'39"W | 477 |
| 38 Los Roques | 28°31'46"N 16°11'38"W | 225 |
| 39 Los Silos | 28°22'33"N 16°48'29"W | 7 |
| 40 Masca | 28°18'44"N 16°50'43"W | 736 |
| 41 Mirador Piedra la Rosa | 28°20'28"N 16°31'31"W | 1482 |
| 42 Montaña de Joco | 28°21'54"N 16°27'52"W | 1955 |
| 43 Fuente los Frailes | 28°26'05"N 16°26'60"W | 639 |
| 44 Playa del Socorro | 28°23'38"N 16°36'17"W | 26 |
| 45 Punta del Sol | 28°27'12"N 16°28'20"W | 49 |
| 46 Puertito de Guimar | 28°17'53"N 16°22'22"W | 13 |
| 47 Puerto de la Cruz | 28°24'47"N 16°32'54"W | 83 |
| 48 Puerto de Santiago | 28°14'23"N 16°50'37"W | 27 |

| | | |
|---|---|---|
| 49 Roque del Conde | 28°06'14"N 16°41'55"W | 991 |
| 50 San Miguel de Abona | 28°06'19"N 16°36'39"W | 658 |
| 51 San Cristóbal de La Laguna | 28°28'11"N 16°18'16"W | 443 |
| 52 Santa Cruz De Tenerife | 28°27'10"N 16°15'23"W | 14 |
| 53 Santiago del Teide | 28°17'44"N 16°48'54"W | 927 |
| 54 Tabaiba | 28°24'08"N 16°19'46"W | 0 |
| 55 Tamaimo | 28°16'08"N 16°49'08"W | 578 |
| 56 Tegueste | 28°31'22"N 16°20'44"W | 356 |
| 57 Vilaflor | 28°09'34"N 16°38'12"W | 1422 |

**La Gomera**

| | | |
|---|---|---|
| 58 Aeropuerto de La Gomera | 28°01'55"N 17°12'38"W | 218 |
| 59 Agulo | 28°11'11"N 17°11'46"W | 199 |
| 60 Alajeró | 28°03'26"N 17°13'50"W | 748 |
| 61 Alojera | 28°09'51"N 17°19'49"W | 122 |
| 62 Alto de Garajonay | 28°06'35"N 17°14'54"W | 1486 |
| 63 Barranco de Argaga | 28°05'04"N 17°19'16"W | 105 |
| 64 Barranco del Cedro | 28°07'27"N 17°13'28"W | 971 |
| 65 Casas Rurales Los Manantiales | 28°06'50"N 17°16'20"W | 1102 |
| 66 Cerco de Armas | 28°09'18"N 17°18'39"W | 603 |
| 67 El Cedro | 28°08'17"N 17°13'01"W | 884 |
| 68 El Molinito | 28°06'46"N 17°08'03"W | 160 |
| 69 El Palmar | 28°09'37"N 17°09'59"W | 353 |
| 70 Epina | 28°09'51"N 17°17'56"W | 762 |
| 71 Hermigua | 28°09'42"N 17°11'53"W | 146 |
| 72 La Dama | 28°03'52"N 17°17'42"W | 413 |
| 73 La Gerode | 28°07'45"N, 17°18'59"W | 910 |
| 74 Las Hayas | 28°07'44"N, 17°18'06"W | 914 |
| 75 LG roadside | 28°07'43"N 17°10'23"W | 514 |
| 76 Mirador de El Palmarejo | 28°07'10"N 17°18'56"W | 708 |
| 77 Mirador de Igualero | 28°05'57"N 17°15'17"W | 1317 |
| 78 Mirador de Tajaqué | 28°06'32"N 17°13'24"W | 1307 |
| 79 Playa Santiago | 28°01'41"N 17°11'56"W | 33 |
| 80 Mirador El Bailadero | 28°07'24"N 17°12'31"W | 1005 |
| 81 San Sebastián de La Gomera | 28°05'30"N 17°06'52"W | 3 |
| 82 Santa Ana | 28°01'41"N 17°12'11"W | 130 |
| 83 Tagamichemt. | 28°05'58"N 17°10'44"W | 976 |
| 84 Teselinde, Arguamull env. | 28°11'44"N 17°17'20"W | 765 |
| 85 Valle Gran Rey | 28°05'17"N 17°20'10"W | 7 |
| 86 Vallehermoso | 28°10'44"N 17°15'52"W | 169 |
| 87 Zona Recreativa Laguna Grande | 28°07'36"N 17°15'25"W | 1249 |
